# Gastrointestinal parasites in non-human primates in zoological institutions in France[Fn FN1]

**DOI:** 10.1051/parasite/2022040

**Published:** 2022-09-20

**Authors:** Irène Vonfeld, Thibaut Prenant, Bruno Polack, Jacques Guillot, Benoît Quintard

**Affiliations:** 1 Parc Zoologique et Botanique de Mulhouse 51 rue du jardin Zoologique 68100 Mulhouse France; 2 Clinique vétérinaire du Coq à l’Ane 24 Boulevard du Commandant René Mouchotte 64320 Bizanos France; 3 Ecole nationale vétérinaire d’Alfort, Biopôle Alfort, Parasitology-Mycology 94704 Maisons-Alfort France; 4 Oniris, Dermatology-Parasitology-Mycology 44300 Nantes France; 5 IRF Research Group, SFR ICAT, University of Angers 4 rue de Larrey 49933 Angers France

**Keywords:** Coproscopy, Digestive parasites, Non-human primates, Diagnosis

## Abstract

Gastrointestinal parasites are frequently encountered in captive non-human primates and infestation may have severe consequences on the animal’s health status. Most of these parasites are also transmissible to humans. Nevertheless, little is known about the prevalence and monitoring modalities of gastrointestinal parasitoses in non-human primates housed in zoological institutions and there are currently no guidelines available for their detection and identification. The objective of this study was to identify the main gastrointestinal parasites that may be observed in non-human primates in zoological institutions in France, as well as to develop a decision-making tree to ease their identification. Twenty-four zoological institutions were surveyed, most of which performed fecal examinations routinely on their non-human primates (91.7%). Most institutions used flotation enrichment protocols to detect gastrointestinal parasites (95.2%) and nematodes were the most frequently encountered parasites (73.0%). A total of 252 fecal samples corresponding to 68 different non-human primate species from these institutions were analyzed using sedimentation and flotation protocols. Protozoa (47.3%) were found to be more frequent than helminths (15.6%). Furthermore, old-world monkeys exhibited a higher parasite load (93.6%) than any other non-human primate species category. Compiled data from fecal examinations allowed the development of a decision-making tree and diagnostic atlas to facilitate parasite diagnosis in captive non-human primates.

## Abstract (fr)

Les parasites gastro-intestinaux sont fréquemment rencontrés chez les primates non-humains en captivité et peuvent avoir des conséquences graves sur l’état de santé de l’animal. La plupart sont également transmissibles à Homme. Néanmoins, on sait peu de choses sur la prévalence et les modalités de surveillance des parasitoses gastro-intestinales chez les primates non-humains hébergés dans des institutions zoologiques et il n’existe actuellement aucune ligne directrice disponible pour leur détection et leur identification. L’objectif de cette étude était d’identifier les principaux parasites gastro-intestinaux pouvant être observés chez les primates non-humains dans les institutions zoologiques françaises, ainsi que de développer un arbre décisionnel pour faciliter leur identification. Vingt-quatre institutions zoologiques françaises ont été interrogées : la plupart (91,7 %) effectuaient des examens fécaux en routine sur leurs primates non-humains. La plupart des établissements utilisaient des protocoles d’enrichissement par flottation pour détecter les parasites gastro-intestinaux (95,2 %) et les nématodes étaient les parasites les plus fréquemment rencontrés (73,0 %). Un total de 252 échantillons fécaux correspondant à 68 espèces différentes de primates non-humains provenant de ces institutions ont été analysés à l’aide de protocoles de sédimentation et de flottation. Les protozoaires (47,3 %) étaient plus fréquents que les helminthes (15,6 %). De plus, les singes de l’ancien monde présentaient une charge parasitaire plus élevée (93,6 %) que toute autre catégorie d’espèces de primates non-humains. Les données compilées des examens fécaux ont permis le développement d’un arbre décisionnel et d’un atlas diagnostique pour faciliter le diagnostic des parasites chez les primates non-humains en captivité.

## Introduction

Captive non-human primates (NHPs) are highly susceptible to gastrointestinal parasitoses [1, 2, 7, 9, 12, 13, 15, 16, 21, 24, 27], with prevalence ranging from 22% [21] to 100% [7] according to the origin of the animals, as well as parasite and host species examined. Parasites with a direct life cycle, like most species of protozoa and helminths, are more prevalent in captive settings as the maintenance of animals in confined living spaces offers them optimum conditions for development and transmission. A positive correlation between parasite richness and host density has been demonstrated in NHPs [11]. Other factors, such as the frequent movement of animals between institutions and other stress-associated situations predispose captive NHPs to parasitoses.

In captive NHPs, gastrointestinal parasites are often responsible for diarrhea and dehydration [2, 16, 27]. *Ancylostoma duodenale*, *Necator americanus*, *Ternidens diminutus* and *Entamoeba histolytica* may cause anemia and tissue damage which may lead to spontaneous abortion and congenital malformation in more severe cases [2, 4, 16, 17, 26, 27].

Some gastrointestinal parasitoses are zoonotic [2, 16, 27]. In particular, molecular-based surveys demonstrated the transmission of protozoa from NHP to humans in European zoological parks [10, 11, 25].

For routine screening for gastrointestinal parasites in zoological institutions, different techniques are used, the most frequent one being morphological identification by microscopic examination with or without an enrichment step by flotation or sedimentation. Specific stains like Lugol or modified Ziehl–Neelsen are also frequently used for further diagnosis. Most of these techniques are easy to perform and inexpensive. Nevertheless, they are labor-intensive, require well-trained microscopists and may lack sensitivity [11]. Moreover, there are currently no complete diagnostic guidelines available to facilitate morphological identification of NHP gastrointestinal parasites. Over the past decade, molecular methods improved diagnostic performance and allowed differentiation of pathogenic species and genotypes circulating in a given host species [11], but these methods are not always applicable to field conditions and are much more expensive than conventional microscopic methods. The first objective of this study was to collect information about gastrointestinal parasites in NHPs in zoological institutions in France and about the techniques used for their detection. The second objective was to develop a decision-making tree and a diagnostic atlas to facilitate the identification of gastrointestinal parasites in captive NHPs.

## Materials and methods

### Questionnaire on gastrointestinal parasites and diagnostic modalities

A detailed questionnaire was designed to evaluate: (1) the use of fecal examination as a preventive or diagnostic method and the modalities of gastrointestinal parasite diagnosis in these institutions; (2) the main parasites encountered in these institutions (see Supplementary data I). The questionnaire was distributed by email to members of the French-speaking Association of Zoological Veterinarians (AFVPZ) on January 29, 2017. At the end of the questionnaire, a request to send feces of NHPs was presented for prospective determination of parasite load in these samples at the Veterinary College of Alfort (EnvA, Maisons-Alfort, France).

### Fecal sampling of non-human primates

Individual and grouped fecal samples were collected from 18 of the 24 zoological institutions that agreed to participate in the prospective study. These samples were identified with the following information: contact details of the institution and corresponding veterinarian; individual or group characteristics; parasitic background; and last deworming treatment. Refrigerated fecal samples were transported to the parasitology laboratory of EnvA (Maisons-Alfort, France) within 3–5 working days of collection, where they were stored in a refrigerator at 4 °C before examination. The analysis was performed within a week after reception of samples. Samples were examined both macroscopically, to verify the presence of nematodes or cestodes, and microscopically after both sedimentation and flotation enrichment. Biosecurity measures such as the use of dispensable gloves and facemasks were taken when handling samples to avoid potential transmission of zoonotic pathogens.

### Sedimentation enrichment protocol

Spontaneous sedimentation enrichment was performed on all received samples. One gram of feces was mixed with 10 mL of 10% formalin to obtain a homogeneous suspension. The suspension was then gauze filtered. Seven millilitres were mixed with 4 mL of diethyl ether, and centrifuged for 5 min at 500 ×*g*. After centrifugation, drops of the pellet were deposited on microscope slides with and without a drop of Lugol stain and at least 10 fields were screened at objective magnification ×10, ×20, ×40 and ×100 successively. This protocol was used to qualitatively identify parasite eggs, cysts and oocysts.

### Flotation enrichment protocol

A flotation enrichment protocol was performed on all samples that contained sufficient feces and samples were analyzed quantitatively by the McMaster technique and qualitatively by the “total” flotation technique. Five grams of feces were mixed with 75 mL of saturated magnesium sulfate (MgSO_4_) aqueous solution to obtain a homogeneous suspension. The suspension was subsequently filtered. Then, a McMaster chamber was filled and left for 10–15 min before being read at objective magnification ×10 to quantitatively assess parasite load in studied samples. In parallel, the flotation solution was poured into a 15 mL tube until a convex dome appeared. A cover slip was then laid flat at the level of the dome and left for 20–25 min, before being withdrawn and mounted on a slide for microscopic screening of at least 10 fields at objective magnification ×10, ×20, ×40 and ×100. This protocol known as the “total” flotation technique was used to qualitatively identify parasite eggs, cysts and oocysts.

### Parasite identification

Morphological diagnosis of parasites was made by a trained microscopist (Thibaut Prenant). Protozoa were identified in their cystic forms and recognized based on the description of those affecting domestic animals and laboratory primates by Euzéby [6], Duszynski et al. [5], Cogswell [3], Strait et al. [26] and Garcia [8]. Helminths were identified according to the morphology of their eggs as described in domestic animals and laboratory primates [3, 6, 8, 26]. Due to the ovo-diagnosis methodology of this study, most helminths like *Bertiella, Ascaris* and *Strongyloides* could only be identified down to genera level. As for strongylids, oxyurids and capillariids, only the family of parasites could be determined. In case of doubtful results, a second trained microscopist (Bruno Polack or Jacques Guillot) screened the sample for a second opinion and final diagnosis was discussed and agreed upon by both observers.

### Data analysis

Fecal samples were used as epidemiological units in this study, as samples could belong to either individuals or group of animals. These were classified into 4 groups: Prosimians (PS), New World Monkeys (NWM), Old World Monkeys (OWM), and Apes (AP). Global infection rates, as well as group infection rates and infection rates according to different types of parasites were calculated. All samples were defined as being independent, as no mixed-species samples were analyzed and considering proper biosecurity measures were carried out in the surveyed institutions. As a result, a *χ*^2^-test was used to compare infection rates between groups. Statistical significance was set at *p* < 0.05.

### Creation of decision-making trees and atlas of common gastrointestinal parasites in captive non-human primates

Parasites and pseudo-parasites identified with the total flotation technique were photographed with a *Nikon Digital Sight DS-Fi1*^®^ lens and analyzed using *Nikon NIS-Elements Basic Research* (*Version 2.30*)^®^ software. These photographs were then classified and used to create decision-making trees allowing the diagnosis of the main species of digestive parasites of captive NHPs using simple fecal screening methods, with links to an atlas of the main parasites affecting captive NHPs.

## Results

### Study population

Twenty-four zoological institutions participated in the study: 22 zoological parks and 2 research centers. A total of 68 non-human primate species were identified within the participating institutions, with 15 PS, 24 NWM, 21 OWM and 8 AP species, respectively (see Supplementary data II). The most represented species were ringed-tailed lemurs (*Lemur catta*), emperor tamarins (*Saguinus imperator*), and red-ruffed lemurs (*Varecia rubra*). From this study population, 252 samples were received for analysis between July 2017 and March 2018, 243 of which were analyzed. In all, 37% of these samples were from NWM, 26% from PS, 19% from OWM, and 18% from AP.

### Modalities of parasite diagnosis in surveyed institutions

Twenty-two of the 24 participating institutions (91.7%) performed fecal examinations on their NHP collection. 95.2% of the institutions performed these tests as part of a preventive screening program. Other situations were identified by zoological parks as reasons to explore for gastrointestinal parasites, such as digestive clinical signs, confirmed parasitism in an animal of the group, treatment follow-up examinations, and pre-transfer examinations.

The most frequently used protocol for parasite screening was identified to be flotation enrichment (95.2%), followed by direct examination (66.7%), coproculture (38.1%), and Baermann technique (9.5%). Formalin-ether sedimentation enrichment techniques, as well as Ritchie’s and Bailenger’s enrichment methods were not often used as diagnostic protocols in the surveyed institutions (4.8% of institutions using each of these). Most frequently used stains included Lugol (4.8% of institutions) and MIF (4.8% of institutions), but these were not used during routine fecal screenings in surveyed institutions. Moreover, only 9.5% of surveyed institutions used molecular techniques to identify specific parasite species.

In most institutions, fecal examinations were performed by veterinarians (95.2%). Veterinary students (33.3%) and zookeepers (4.8%) carried out screenings to a lesser extent, and their results were reviewed by veterinarians in 89.5% of positive cases. Fewer than half of the surveyed institutions (47.6%) reported sending the samples for external laboratory analysis.

### Infection background in surveyed institutions

According to surveyed zoological institutions, captive NHP species seem to be more affected by helminths (73.0%) than protozoa (27.0%). Helminth infections in these institutions were caused by a variety of species including trichurids, capillariids, strongylids (*Molineus* and *Angiostrongylus* sp.), spirurids (*Streptopharagus* and *Physaloptera* sp.), *Ascaris* sp., *Strongyloides* sp., and cestodes (*Taenia* and *Hymenolepis* sp*.*)*.* No trematode infection was reported by any institution. Amongst protozoa, coccidia (*Cryptosporidium, Eimeria, Isospora* and *Cyclospora* sp*.*) seemed to be the most frequent. Other protozoa reported by surveyed institutions were ciliates (*Balantioides coli*), flagellates (*Giardia intestinalis, Retortamonas intestinalis* and *Trichomonas intestinalis*), and amoeba (*Entamoeba* sp*.*).

### Prevalence and identification of gastrointestinal parasites in captive non-human primates

**Figure 1:**
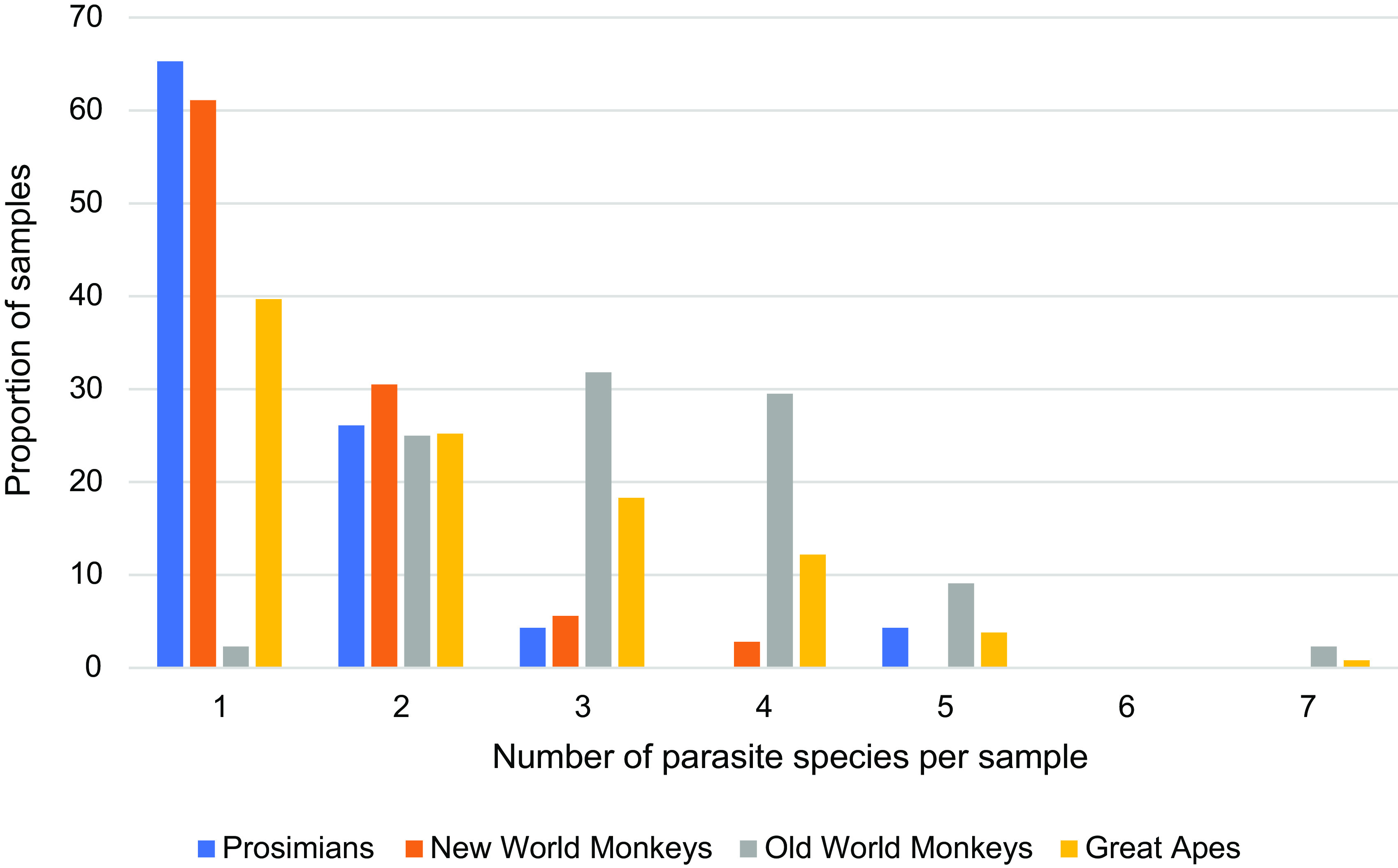
Distribution of multispecies parasitism in non-human primate groups.

Prospectively analyzed samples belonged to the same species as reported in the questionnaires and were therefore considered to be representative of the NHP population in zoological institutions in France. Of these samples, 131/243 (53.9%) were positive to at least one parasite species. OWM were the most affected group of primates, with a prevalence of gastrointestinal parasites of 93.6%, followed by AP (63.6%). NWM and PS were the least affected groups, with prevalence of 40.0% and 37.1%, respectively. Parasite infection rates are summarized in Table 1. In all NHP categories, protozoa were more common than helminths. Moreover, global infection rates, as well as both helminth and protozoa infection rates were statistically higher in OWM than in other groups (global infection rate: *p*_OWM>PS_ = 2.646 × 10^−9^, *p*_OWM>NWM_ = 0.005; helminths infection rate: *p*_OWM>PS_ = 0.007, *p*_OWM>NWM_ = 0.018; protozoa infection rate: *p*_OWM>PS_ = 7.411 × 10^−19^, *p*_OWM>NWM_ = 6.43 × 10^−10^; *p*_OWM>AP_ = 0.0004). Infection by multiple parasites was identified in 60.3% of the samples, and up to 7 different species of parasites could be identified in a single sample. Mixed infections were more frequently found in OWM than in any other groups (Fig. 1).

**Table 1:** Overall and group-specific global, helminth and protozoa infection rates.

Group	Global infection rate (%)	Helminth infection rate (%)	Protozoa infection rate (%)
Prosimians	37.1^a^	8.1^a^	30.6^a^
New world monkeys	40.0^a^	11.1^a^	34.4^a^
Old world monkeys	93.6^b^	29.8^c^	91.5^b^
Apes	63.6^b^	20.5^b^	56.8^b^
Global	53.9	15.6	47.3

a Infection rates not significantly different between groups (*p* < 0.05).

b Infection rates not significantly different between groups identified with a “b” but significantly greater than groups identified with an “a” and significantly lower than those identified with a “c” (if applicable) (*p* < 0.05).

c Infection rates significantly greater than groups identified with an “a” or “b” (*p* < 0.05).

**Figure 2:**
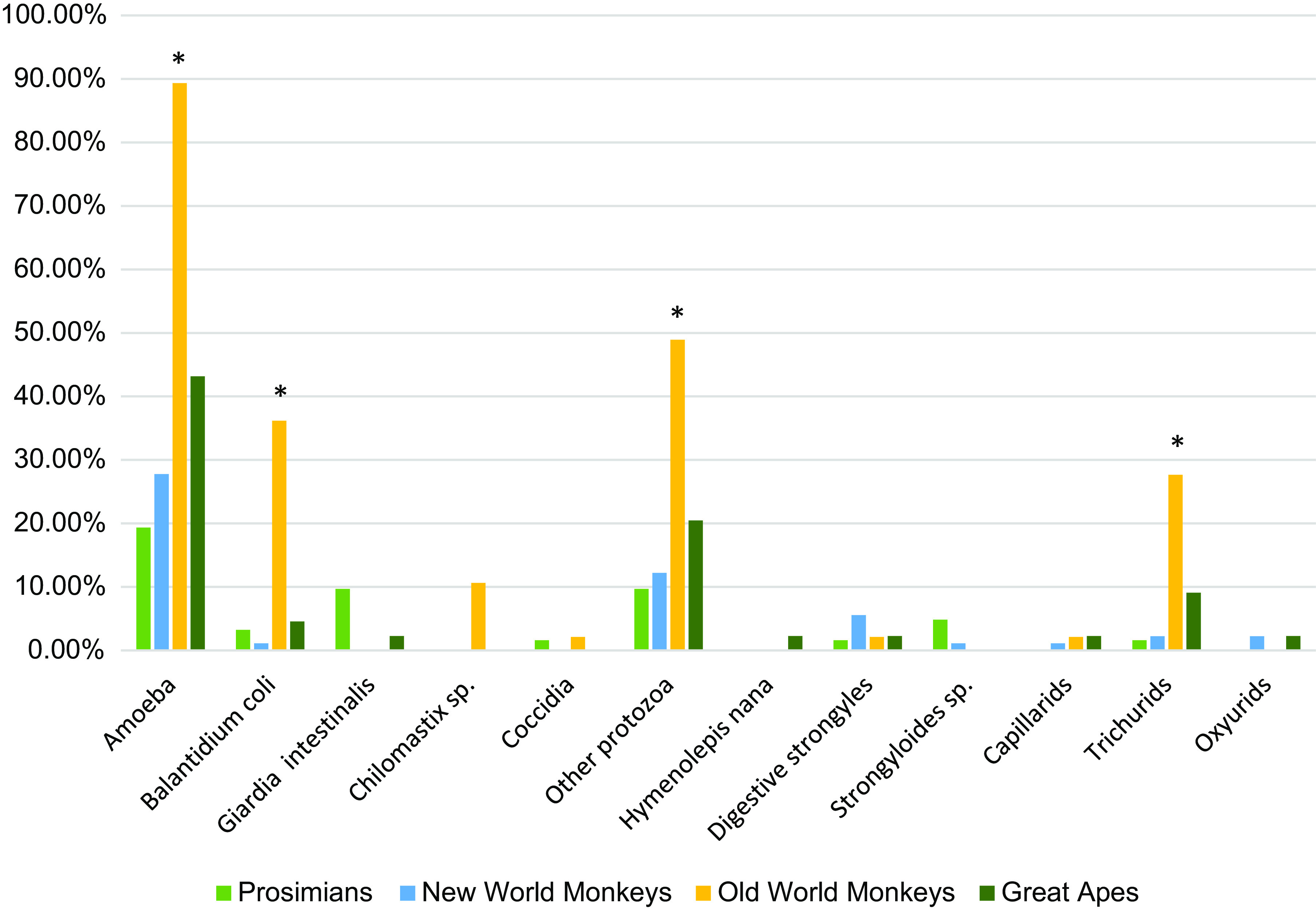
Infection rates of different parasites according to host species group. ^*^Group infection rate significantly greater than all other groups for the parasite of interest (*p* < 0.05).

Different helminths and protozoa were identified during the prospective study by either sedimentation or flotation protocols (Fig. 2). Protozoan species can be further classified in different taxa: amoeba, flagellates, coccidia and ciliates. Some parasites were classified down to genera and species level. Among the amoeba identified, 3 genera were differentiated, but were not classified down to species level: *Entamoeba, Iodamoeba* and *Endolimax*. These were shown to be significantly more frequent in OWM than in PS (*p*_OWM>PS_ = 1.838 × 10^−12^), NWM (*p*_OWM>NWM_ = 2.636 × 10^−11^), and AP (p_OWM>AP_ = 8.845 × 10^−8^). AP were more affected by amoeba than PS (*p*_AP>PS_ = 0.013). Among the flagellates identified, *Giardia intestinalis* and *Chilomastix* sp. were differentiated. *Giardia intestinalis* was the only parasite group to be more frequent in PS than in OWM (*p*_PS>OWM_ = 0.036) and NWM (*p*_PS>NWM_ = 0.004). *Chilomastix* sp*.* was significantly more frequent in OWM than in PS (*p*_OWM>PS_ = 0.013) and NWM (*p*_OWM>NWM_ = 0.004). Coccidia were not differentiated down to genus level. Finally, the only diagnosed ciliate was *Balantioides coli*. It was shown to be more frequent in OWM than in any other groups (*p*_OWM>PS_ = 2.285 × 10^−5^; *p*_OWM>NWM_ = 3.792 × 10^−8^; *p*_OWM>AP_ = 0.001). Other small protozoa of species like *Enteromonas hominis* and *Retortamonas intestinalis*, classified in the category called “Other protozoa” in the present study due to their small size and non-specific aspect, also affected OWM more than any other group (*p*_OWM>PS_ = 1.216 × 10^−5^; *p*_OWM>NWM_ = 6.346 × 10^−6^; *p*_OWM>AP_ = 0.009).

Helminth species were either cestodes, with only one species identified (*Hymenolepis nana*), or nematodes. Amongst nematodes, different groups of parasites were identified: strongylids, *Strongyloides* sp., capillariids, trichurids and oxyurids. The only statistical difference of helminth infection rates between groups concerned trichurid infection, which was more frequent in OWM than in any other group (*p*_OWM>PS_ = 0.0002; *p*_OWM>NWM_ = 2.250 × 10^−5^; *p*_OWM>AP_ = 0.045).

### Decision-making tree and atlas for the diagnosis of gastrointestinal parasites in captive non-human primates

**Figure 3:**
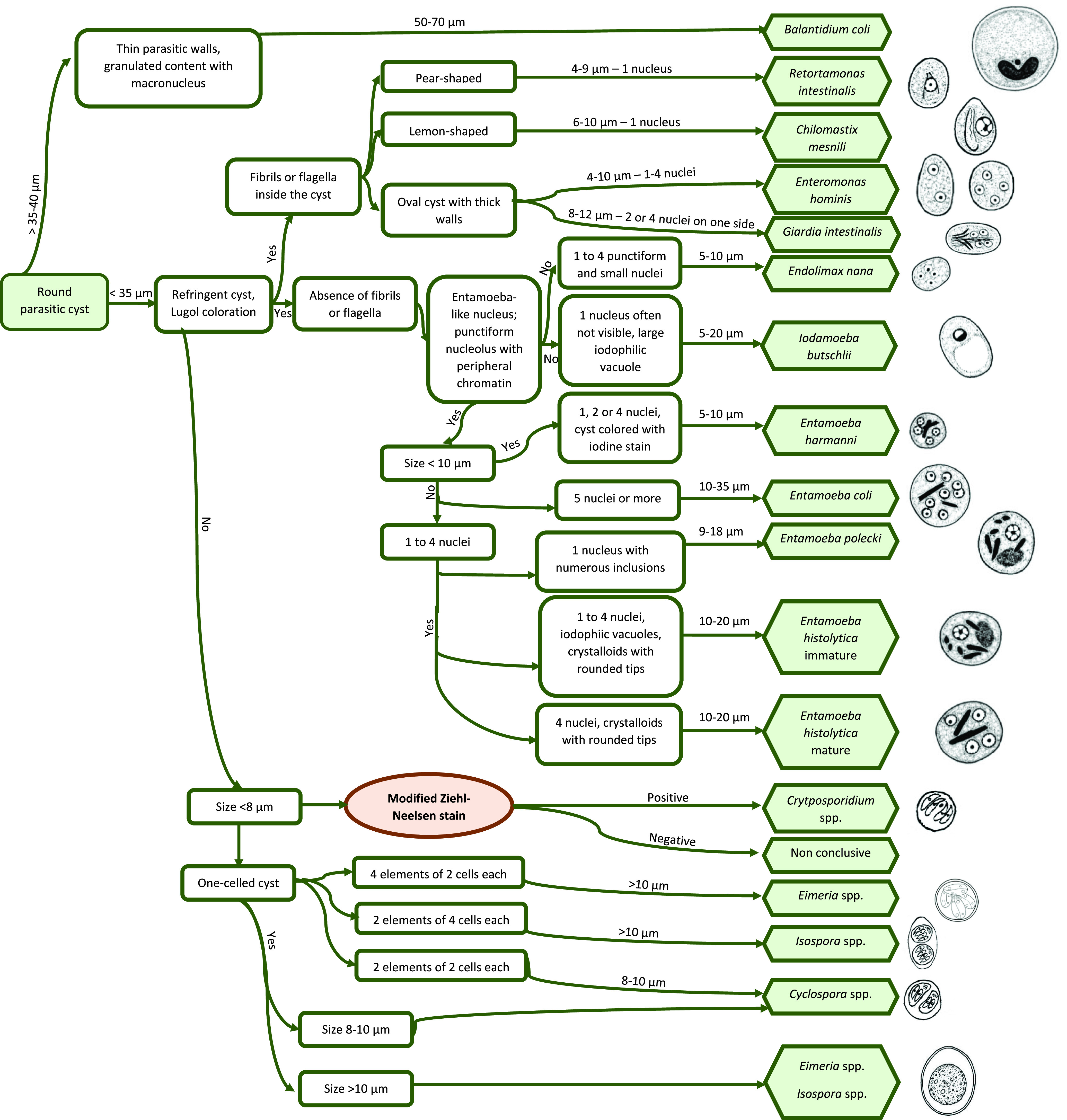
Decision-making tree for the diagnosis of protozoa in non-human primates.

**Figure 4:**
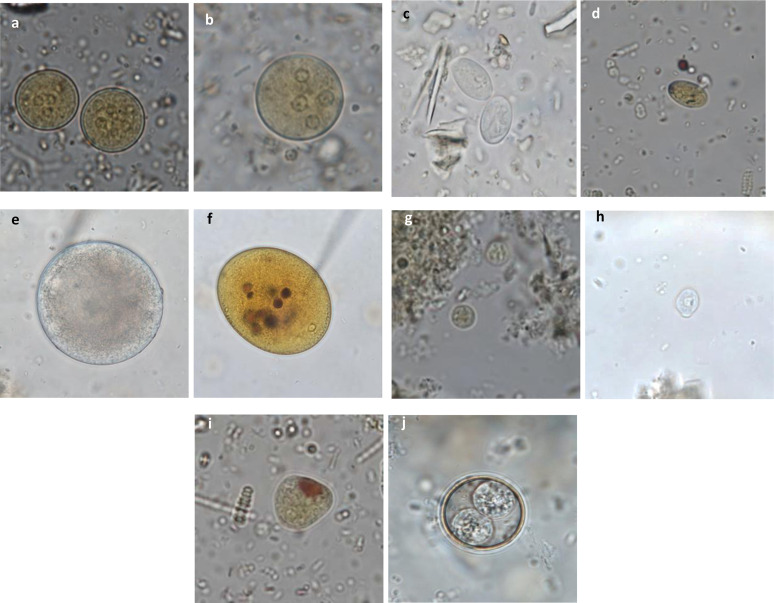
Diagnostic atlas for the identification of protozoan cysts in non-human primates. (a) *Entamoeba* sp. cysts stained with Lugol (×100): eccentric nuclei with a voluminous endosome and granulomatous peripheral chromatin; (b) *Entamoeba* sp. cyst stained with Lugol (×100); (c) *Giardia intestinalis* cysts (×100): oval cysts of 8–12 μm with a thin outer membrane and 2–4 nuclei and a flagellum; (d) *Giardia intestinalis* cyst stained with Lugol (×100); (e) *Balantioides coli* cyst (×100): spherical to ovoid cyst of 50–70 μm in diameter with a thick membrane, granular content, one macro- and one micronucleus; (f) *Balantioides coli* stained with Lugol (×100); (g) *Endolimax* sp. cyst stained with Lugol (×100): small round cysts with a thin outer membrane and 1–4 punctiform nuclei with voluminous, irregular endosomes without perisomes; (h) *Chilomastix* sp. cyst (×100): small piriform cysts with a thin and refringent membrane, one nucleus and a cytostome containing the flagellum; (i) *Iodamoeba* sp. cyst stained with Lugol (×100): oval cysts with a small round nucleus containing a large vacuole, and a peripheral iodophilic voluminous vacuole; (j) *Isospora* sp. sporulated oocyst (×100): round cysts containing 2 sporocysts with 4 sporozoites each.

**Figure 5:**
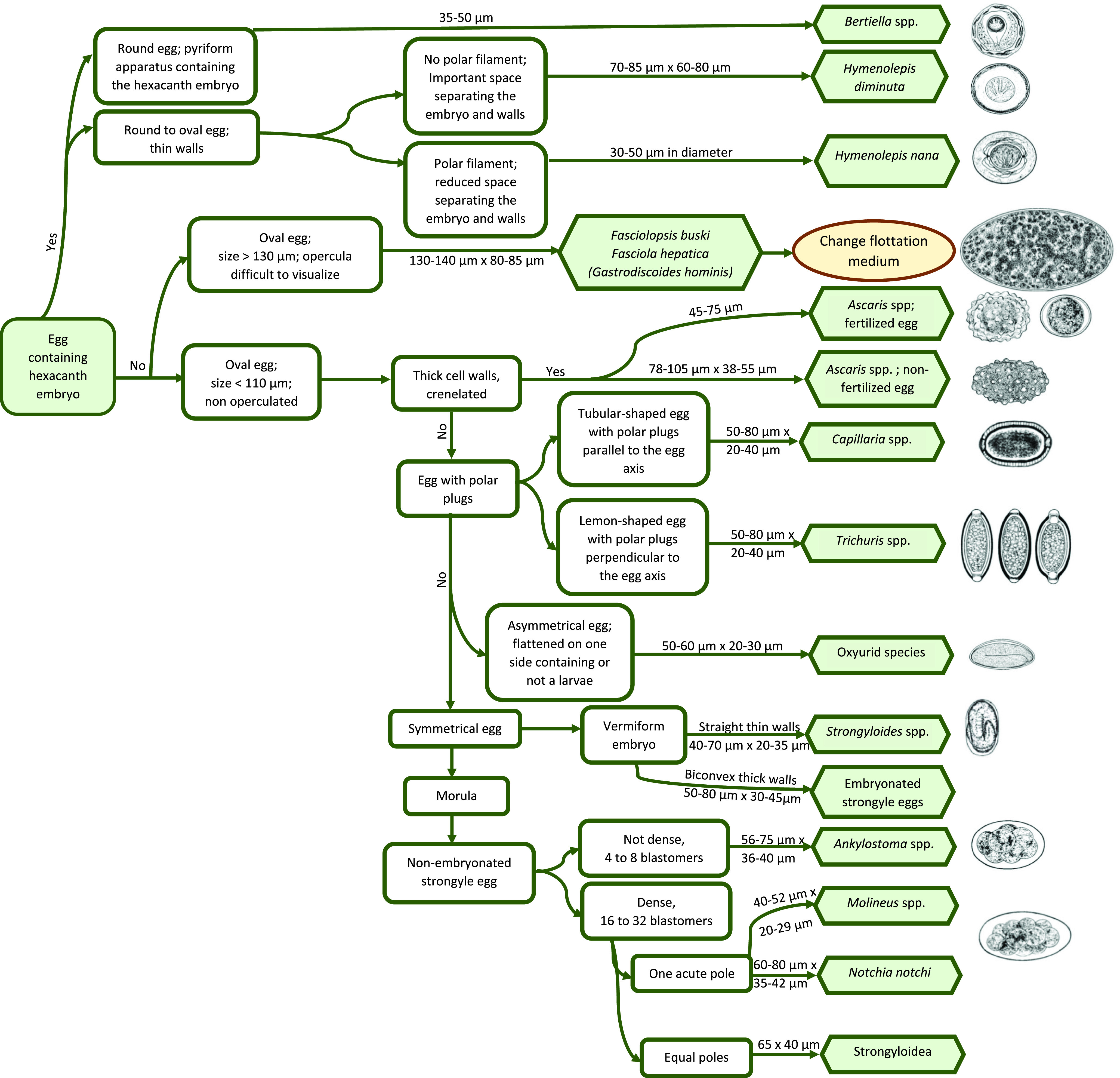
Decision-making tree for the diagnosis of helminths in non-human primates.

**Figure 6:**
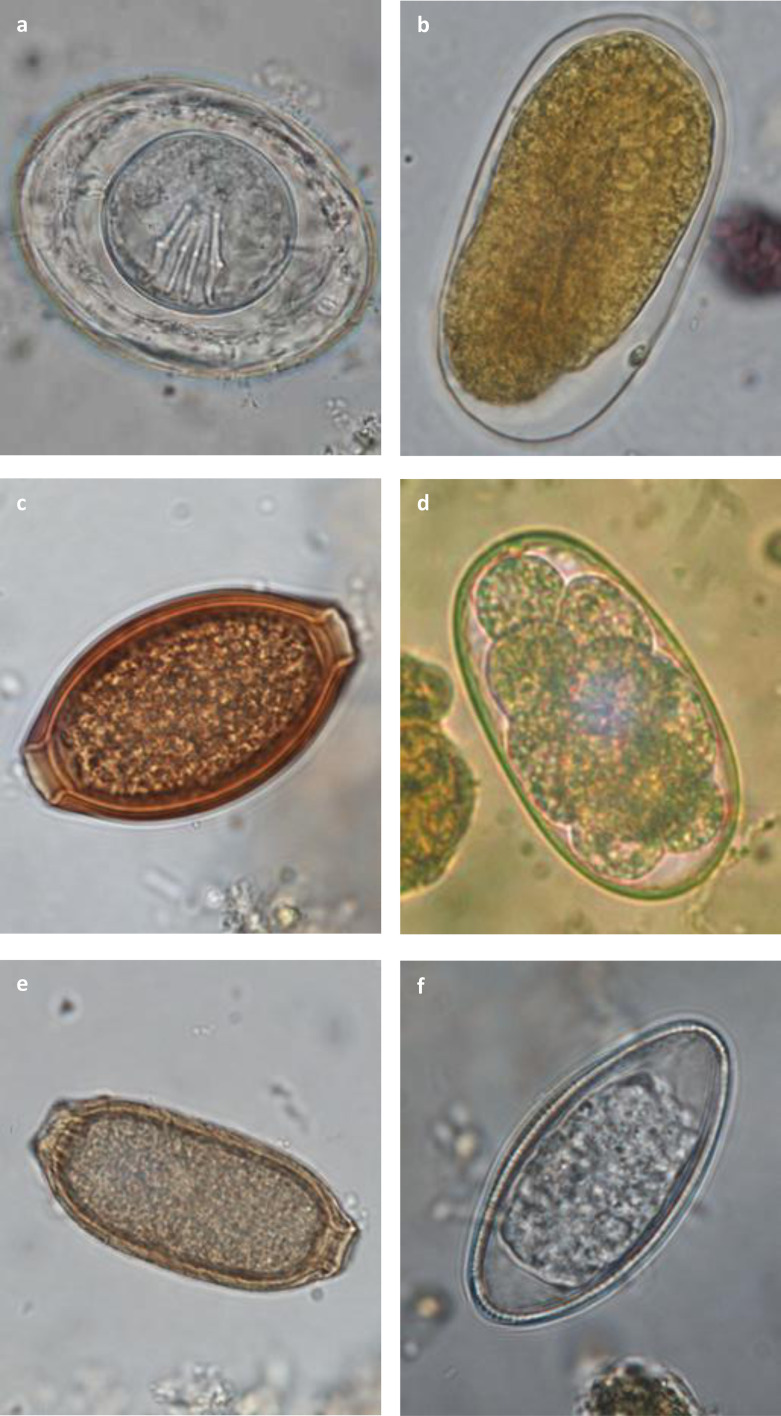
Diagnostic atlas for the identification of helminthic eggs in non-human primates. (a) *Hymenolepis nana* egg (×100): round to oval of 30–50 μm in diameter containing an hexacanth embryo with equatorial polar filaments; (b) *Strongyloides* sp. egg (×100): oval, symmetrical of 40–70 μm by 20–35 μm with a thin membrane and parallel lateral sides; larvae can be present inside the egg; (c) Trichurid egg (×100): oval, symmetrical of 50–80 μm by 20–40 μm with a smooth outer membrane and polar plugs striated perpendicular to the axis of the egg; (d) Strongylid egg (×100): oval of variable size with a thick outer membrane, asymmetrical lateral sides and containing a morula; (e) Capillariid egg (×100): oval, symmetrical of 50–80 μm by 20–40 μm with a smooth outer membrane and polar plugs striated parallel to the axis of the egg; (f) Oxyurid egg (×100): oval, slightly asymmetrical of 50–60 μm by 20–30 μm with one flat lateral side, a thick outer membrane and a vermiform embryo.

All the acquired data mentioned above allowed the elaboration of a decision-making tree and atlas for the diagnosis of protozoa (Figs. 3 and 4) and helminths (Figs. 5 and 6) in captive NHPs. These documents, along with information regarding the parasites mentioned, can be found on a website designed for the purpose (http://www.zoo-mulhouse.com/atlas-parasites-primates).

## Discussion

In the present prospective study, 53.6% of examined groups were infected with at least one parasite species. No trematode infection was identified. These results are consistent with previously published data on NHP parasitism in European zoological institutions, in which prevalence ranges from 22% to 100% [7, 21].

Protozoan infections (47.3%) were more prevalent than helminth infections (15.6%), which supports the findings of previous European NHP fecal screening studies as well [7, 13, 21], although the prevalence of protozoan infection was slightly lower in our study. This can be due to new biosecurity measures and higher efficacy of prevention and treatment protocols established in zoological institutions over the past few years. Recently published molecular-based reports of prevalence of protozoa indicated infection rates in NHP ranging from 41.0% [12] to 62.7% [11]. The higher prevalence of protozoa as compared to helminths can be explained by the simplicity of their life cycle: most protozoa have a direct life cycle with a short prepatent period. Protozoa are often infective directly upon excretion and require a low dose for infection.

Unlike the results of the present study, some publications reported a higher prevalence of helminths than protozoa in captive NHP [1, 7, 12, 17]. For instance, Fagiolini et al. [7] reported that in one of the studied zoological parks, nematodes were more prevalent than protozoa in yellow baboons (*Papio cyanocephalus*). This can be explained by the different epidemiological setting of the institution in question, a safari park characterized by large open natural enclosures, which could favor the development of parasites with indirect life cycles and be similar to what would occur in the wild. Similarly, a study comparing parasite load in captive and wild-trapped African NHP showed that captive NHP had a higher protozoan parasitic load than wild free-ranging ones and wild-trapped animals were more affected by cestodes and nematodes with more complex life cycles [18].

In this study, OWM were statistically more affected by both protozoa and helminths than any other NHP category. Amoeba, *Balantioides* sp., other protozoa and trichurids were significantly more prevalent in this group. This is similar to what has been described in previous studies [13]. One explanation for this phenomenon would be that most OWM are more ground-dwelling than NWM and PS species [20, 23]. Therefore, they may be more often in contact with contaminated soil. A tendency for arboreal primate species to be less affected by gastrointestinal parasites has been reported in various publications [15, 17, 19]. Munene et al. [19] even showed that *Strongyloides fuelleborni* was absent in animals housed in cages hanging above the floor, whereas it was frequent in animals housed in floor cages. In the same study, Sykes monkeys (*Cercopithecus mitis*) were less infected by parasites than olive baboons (*Papio cyanocephalus anubis*) probably because of their arboreal nature [19]. Other studies have hypothesized that an arboreal lifestyle would prevent parasite contamination [15, 17]. It would thus be interesting to compare parasite load between different species of captive OWM of arboreal and terrestrial lifestyle in order to confirm this hypothesis.

One of the limitations of the present study was the low statistical significance of the quantitative data of the parasite loads observed. Most fecal samples were insufficient for quantitative analysis, and when it was possible, fecal parasite load was most often too low for analysis. Moreover, species which had their samples analyzed quantitatively were not representative of the PNH population studied and these results were therefore excluded from the study. The quantitative analysis of gastrointestinal parasites is important, as it can be a determining factor in the decision whether to treat an animal or group of animals. Antiparasitic treatments need to be well thought-out in order to avoid resistance development [2, 16, 17, 27] and one of the ways to determine whether to treat an animal is to consider its parasitic load. For instance, the World Health Organization established infection intensity categories for *Trichuris trichiura* for humans to help the management of large-scale deworming programs [28]. Aviruppola et al. [1] showed that of 7-primate species affected by trichurids, only Hamadryas baboons (*Papio hamadryas*) had a count higher than 1000 epg and would thus require anthelmintic treatment. In the present study, clinical signs at the time of sampling were not reported. Therefore, no conclusions can be given regarding the pathogenicity of the parasites identified. In further investigations, combined clinical data with quantitative parasite load determinations could allow the development of species and parasite-specific thresholds for treatment. Antiparasitic treatment should always be coupled to biosecurity and hygiene measures when a parasitic infection is diagnosed in a group. Most parasites develop due to the confinement of animals at high density. Zootechnical measures like improved husbandry procedures and disease preventive measures, dung removal, routine monitoring of parasitic diseases and the use of selective treatment were effective in reducing parasite load in a zoological park in Italy [7] and should be considered the foundation of parasite prevention in zoological institutions. As most parasites affecting NHPs have been shown to be zoonotic [2, 16, 24, 27], with potential transmission from animals to keepers of protozoa like *Giardia intestinalis, Cryptosporidium hominis* and *Blastocystis* sp. confirmed by molecular based surveys in European zoos [10, 11], it is of upmost importance to respect strict hygiene protocols in order to limit the risk of transmission to the staff and visitors. Another limitation of the present study is a possible underestimation of some parasite prevalence due to the lack of sensitivity of diagnostic modalities used during fecal examination. Samples were not systematically stained with modified Ziehl–Neelsen coloration, which could have induced a bias in the diagnosis of certain parasite species like *Cryptosporidium* sp. No molecular diagnostic techniques were used in our study, and this probably accounts for the absence of certain parasite species which are difficult to diagnose microscopically but have been shown to have a high prevalence in NHPs in previous studies. For instance, *Blastocystis* DNA has been detected in 20.3% of NHP fecal samples from five zoological institutions in Spain, France, and Germany [10]. Molecular diagnosis could also have allowed the differentiation of *Entamoeba* from other amoeba species, as well as the identification of the different species within the genus. Captive NHPs are suitable hosts for several *Entamoeba* species, namely *E. bangladeshi, E. chattoni, E. coli, E. dispar, E. ecuadoriensis, E. hartmanni, E. histolytica, E. moshkovskii, E. nutalli* and *E. polecki* [11, 14, 22] which are all morphologically identical. However, the species show different virulence capabilities, with *E. histolytica* being the most pathogenic and zoonotic [11], and *E. dispar* being the most prevalent but non-pathogenic.

Underestimation of gastrointestinal parasite prevalence due to lack of sensitivity of diagnostic modalities is even more obvious for onsite diagnosis in surveyed institutions, especially for protozoa identification. The experience of the person performing fecal examinations could also have played a role in the lack of sensitivity. In some of the surveyed institutions, fecal screenings were performed by veterinary students or zookeepers, who did not systematically receive routine training for microscopic diagnosis of parasites. As protozoa are often smaller and more difficult to diagnose than helminths, they may be underdiagnosed. This probably accounts for the discrepancy in the prevalence of helminths and protozoa when onsite and prospective diagnosis were compared. This also highlights the need for: (1) adoption and implementation of a standardized protocol for the diagnosis of gastrointestinal parasites by conventional methods; (2) routine training of microscopists to guarantee competence and skills, as is intended in the present study with the development of decision-making trees and atlas of the main gastrointestinal parasites in NHP; and (3) implementation of inter-laboratory trials to evaluate the performance of the methods.

## Conclusion

Many gastrointestinal parasites can affect captive NHPs, most of them being nematodes and protozoa. Fecal analyses are regularly performed in most zoological institutions in France, both as part of screening programs as well as secondarily to clinical suspicion. Flotation enrichment techniques are most often used in these institutions, leading to more frequent diagnosis of helminths than other gastrointestinal parasites. Nevertheless, when performing extensive fecal diagnosis, and according to previous investigations [7, 13, 21], protozoa are most frequently encountered. Significant differences between the infection rates of different groups of non-human primate species have been identified, with OWM being more affected than any other primates. These data should be considered when elaborating a screening protocol for gastrointestinal parasitoses in zoological parks. In order to facilitate diagnosis, the decision-making trees and atlas provided in the present article should be used.

## Supplementary materials

Supplementary material is available at https://www.parasite-journal.org/10.1051/parasite/2022040/olm. Supplementary data I: Questionnaire on gastrointestinal parasites in non-human primates of French zoological institutions and their diagnostic modalities. Supplementary data II: List of non-human primate species represented in the study.
