# Supplementary material for: Gastrointestinal parasites in non-human primates in zoological institutions in France
Source: Parasite. 2022 Sep 20;29:43. doi: 10.1051/parasite/2022040 (PMC9487514; doi:10.1051/parasite/2022040)
Supplement: Supplementary data I: — Questionnaire on gastrointestinal parasites in non-human primates of French zoological institutions and their diagnostic modalities. [file parasite-29-43-s1.pdf]

Supplementary data I

Questionnaire on gastrointestinal parasites in non-human primates of French zoological institutions and their diagnostic modalities

GENERAL INFORMATION

Name of the veterinarian:
Name of the institution:
Postal address of the institution:
E-mail address of the veterinarian:
AGREEMENT TO PARTICIPATE TO THE PROJECT (yes/no):

SPECIES OF NON-HUMAN PRIMATES HOUSED IN YOUR INSTITUTION

WHAT SPECIES OF PRIMATES ARE HOUSED IN YOUR INSTITUTION?

- □ *Eulemur cinereiceps*
- □ *Eulemur (macaco) flavifrons*
- □ *Eulemur coronatus*
- □ *Eulemur mongoz*
- □ *Eulemur rubriventer*
- □ *Eulemur fulvus*
- □ *Eulemus macaco*
- □ *Lemur catta*
- □ *Hapalemur alaotrensis*
- □ *Prolemur simus*
- □ *Propithecus coronatus*
- □ *Varecia variegata subcinta*
- □ *Varecia variegata*
- □ *Galago senegalensis*
- □ *Tarsius spp.*
- □ *Lagothrix spp.*
- □ *Saimiri sciureus*
- □ *Saimiri boliviensis boliviensis*
- □ *Saimiri boliviensis peruviensis*
- □ *Saguinus bicolor*
- □ *Saguinus midas*
- □ *Saguinus imperator*
- □ *Saguinus oedipus*
- □ *Callimico goeldii*
- □ *Leontopithecus chrysomelas*
- □ *Leontopithecus rosalia*
- □ *Callithrix jacchus*
- □ *Callithrix geoffroyi*
- □ *Cebuella pygmaea*
- □ *Mico argentatus*
- □ *Ateles hybridus*
- □ *Ateles paniscus*
- □ *Ateles fusciceps rufiventris*
- □ *Pithecia pithecia*
- □ *Chiropotes satanas*
- □ *Sapajus xanthosternos*
- □ *Cebus apella*
- □ *Cebus capucinus*
- □ *Alouatta spp.*
- □ *Chlorocebus spp.*
- □ *Cercopithecus roloway*
- □ *Cercopithecus lhoesti*
- □ *Cercopithecus hamlyni*
- □ *Cercopithecus neglectus*
- □ *Cercopithecus diana*
- □ *Cercopithecus nictitans*
- □ *Allenopithecus nigriviridis*
- □ *Macaca tonkeana*
- □ *Macaca silenus*
- □ *Macaca nemestrina*
- □ *Macaca sylvanus*
- □ *Papio hamadryas*
- □ *Papio papio*
- □ *Papio anubis*
- □ *Theropithecus gelada*
- □ *Lophocebus aterrimus*
- □ *Cercocebus torquatus*
- □ *Mandrillus sphinx*
- □ *Colobus spp.*
- □ *Procolobus spp.*
- □ *Piliocolobus spp.*
- □ *Symphalangus syndactylus*
- □ *Nomascus leucogenys*
- □ *Nomascus siki*
- □ *Nomascus gabriellae*
- □ *Hylobates pileatus*
- □ *Hylobates lar*
- □ *Hylobates muelleri*
- □ *Gorilla gorilla*
- □ *Gorilla beringei*
- □ *Pongo pygmaeus*
- □ *Pongo abelli*
- □ *Pan troglodytes*
- □ *Pan paniscus*
- □ Other

FECAL SCREENINGS IN NON-HUMAN PRIMATES IN YOUR INSTITUTION

ARE FECAL SCREENING PERFORMED IN YOUR NON-HUMAN PRIMATE SPECIES (yes/no):
IF SO, IN WHAT SPECIES?

- □ *Eulemur cinereiceps*
- □ *Eulemur (macaco) flavifrons*
- □ *Eulemur coronatus*
- □ *Eulemur mongoz*
- □ *Eulemur rubriventer*
- □ *Eulemur fulvus*
- □ *Eulemus macaco*
- □ *Lemur catta*
- □ *Hapalemur alaotrensis*
- □ *Prolemur simus*
- □ *Propithecus coronatus*
- □ *Varecia variegata subcinta*
- □ *Varecia variegata*
- □ *Tarsius spp.*
- □ *Lagothrix spp.*
- □ *Saimiri sciureus*
- □ *Saimiri boliviensis boliviensis*
- □ *Saimiri boliviensis peruviensis*
- □ *Saguinus bicolor*
- □ *Saguinus midas*
- □ *Callimico goeldii*
- □ *Saguinus imperator*
- □ *Saguinus oedipus*
- □ *Chlorocebus spp.*
- □ *Cercopithecus roloway*
- □ *Cercopithecus lhoesti*
- □ *Cercopithecus hamlyni*
- □ *Cercopithecus neglectus*
- □ *Cercopithecus diana*
- □ *Cercopithecus nictitans*
- □ *Allenopithecus nigriviridis*
- □ *Macaca tonkeana*
- □ *Macaca Silenus*
- □ *Macaca nemestrina*
- □ *Macaca sylvanus*
- □ *Papio hamadryas*
- □ *Symphalangus syndactylus*
- □ *Hylobates pileatus*
- □ *Nomascus leucogenys*
- □ *Nomascus siki*
- □ *Nomascus gabriellae*
- □ *Hylobates lar*
- □ *Hylobates muelleri*
- □ *Gorilla gorilla*

- ☐ *Galago senegalensis*

- ☐ *Leontopithecus chrysomelas*
- ☐ *Leontopithecus rosalia*
- ☐ *Callithrix jacchus*
- ☐ *Callithrix geoffroyi*
- ☐ *Cebuella pygmaea*
- ☐ *Mico argentatus*
- ☐ *Ateles hybridus*
- ☐ *Ateles paniscus*
- ☐ *Ateles fusciceps rufiventris*
- ☐ *Pithecia pithecia*
- ☐ *Chiropotes satanas*
- ☐ *Sapajus xanthosternos*
- ☐ *Cebus apella*
- ☐ *Cebus capucinus*
- ☐ *Alouatta spp.*

- ☐ *Papio papio*
- ☐ *Papio anubis*
- ☐ *Theropithecus gelada*
- ☐ *Lophocebus aterrimus*
- ☐ *Cercocebus torquatus*
- ☐ *Mandrillus sphinx*
- ☐ *Colobus spp.*
- ☐ *Procolobus spp.*
- ☐ *Piliocolobus spp.*

- ☐ *Gorilla beringei*
- ☐ *Pongo pygmaeus*
- ☐ *Pongo abelli*
- ☐ *Pan troglodytes*
- ☐ *Pan paniscus*
- ☐ Other

METHODOLOGY OF FECAL SCREENINGS

AT WHAT FREQUENCY ARE FECAL SCREENINGS PERFORMED:

UNDER WHAT EPIDEMIOLOGICAL CIRCUMSTANCES ARE THESE SCREENING PERFORMED?

- ☐ As routine diagnosis
- ☐ In case of digestive clinical signs within the group
- ☐ After infection periods
- ☐ In case of confirmed parasitosis in the group
- ☐ Other:

WHICH ANIMALS ARE SAMPLED FOR THESE SCREENINGS

- ☐ Sick individuals
- ☐ Healthy individuals

WHAT PROPORTION OF THE ANIMALS ARE SAMPLED DURING THESE SCREENINGS?

WHAT DIAGNOSTIC METHODS ARE USED DURING THESE SCREENINGS?

- ☐ Lugol staining
- ☐ Merthiolate-Iodine-Formaldehyde staining
- ☐ Modified Ziehl-Neelsen staining
- ☐ Coproculture
- ☐ Direct microscopic examination
- ☐ Flotation enrichment
- ☐ Sedimentation enrichment method
- ☐ Baermann enrichment method
- ☐ Kato enrichment method
- ☐ Ballenger enrichment method
- ☐ Faust enrichment method
- ☐ Ritchie enrichment method
- ☐ Telemann-Rivas enrichment method
- ☐ Willis enrichment method
- ☐ Other (please comment):

PARASITIC BACKGROUND OF YOUR INSTITUTION

WHAT IS THE PARASITIC BACKGROUND OF SCREENED SPECIES? (add parasite species diagnosed if possible)

Species	Amoeba	Flagellates	Coccidia	Cestodes	Oxyurids	Strongylids	Other nematodes
Eulemur cinereiceps							
Eulemur (macaco) flavifrons							
Eulemur coronatus							
Eulemur mongoz							
Eulemur rubriventer							
Eulemur fulvus							
Eulemur macaco							
Lemur catta							
Hapalemur alaotrensis							
Prolemur simus							
Propithecus coronatus							
Varecia variegate subcinta							
Varecia variegata							
Galago senegalensis							

Tarsius spp.							
Lagothrix spp.							
Saimiri sciureus							
Saimiri boliviensis boliviensis							
Saimiri boliviensis peruviensis							
Saguinus bicolor							
Saguinus midas							
Saguinus imperatoru							
Saguinus Oedipus							
Callimico goeldii							
Leontopithecus chrysomelas							
Leontopithecus rosalia							
Callithrix jacchus							
Callithrix geoffroyi							
Cebuella pygmaea							
Mico argentatus							
Ateles hydribdus							
Ateles paniscus							
Ateles fusciceps rufiventris							
Pithecia pithecia							
Chiropotes satanas							
Sapajus xanthosternos							
Cebus apella							
Cebus capucinus							
Alouatta spp.							
Chlorocebus spp.							
Cercopithecus roloway							
Cercopithecus lhoesti							
Cercopithecus hamlyni							
Cercopithecus neglectus							
Cercopithecus diana							
Cercopithecus nictitans							
Allenopithecus nigriviridis							
Macaca tonkeana							
Macaca silenus							
Macaca nemestrina							
Macaca sylvanus							
Papio hamadryas							
Papio papio							
Papio anubis							
Theropithecus gelada							
Lophocebus aterrimus							
Cercocebus torquatus							
Mandrillus sphinx							
Colobus spp.							
Procolobus spp.							
Piliocolobus spp.							
Symphalangus syndactylus							
Nomascus leucogenys							
Nomascus siki							
Nomascus gabriellae							
Hylobates pileatus							
Hylobates lar							

Hylobates muelleri							
Gorilla gorilla							
Gorilla beringei							
Pongo pygmaeus							
Pongo abelli							
Pan troglodytes							
Pan paniscus							

PARASITES IN OTHER SPECIES OF YOUR INSTITUTIONS

ARE THOSE PARASITES FOUND IN OTHER NON-PRIMATE SPECIES IN YOUR INSTITUTION (yes/no):

IF SO, IN WHAT SPECIES?

Amoeba (*Entamoeba, Endolimax, ...*)	
Flagellates (*Giardia, Chilomastix, Enteromonas, …*)	
Blastocystis	
Coccidia (*Cryptosporidium, …*)	
Cestodes (*Bertiella, …*)	
Oxyurids (*Enterobius, …*)	
Strongyles (*Ancylostoma, Ternidens, Oesophagostomum, Trichostrongylus, …*)	
Other nematodes (*Trichuris, Ascaris, Strongyloides, …*)	

DEGREE OF CONFIDENCE

WHO CARRIES OUT FECAL SCREENINGS IN YOUR INSTITUTION

- □ Zoo veterinarians
- □ Zookeepers
- □ Veterinary students
- □ Other:

WHAT IS THE DEGREE OF CONFIDENCE OF DIAGNOSIS (0-100):

IN CASE OF PARASITE IDENTIFICATION OR DOUBTFUL RESULTS, IS THE SAMPLE SCREENED BY A VETERINARIAN FOR SECOND OPINION (yes/no):

PROSPECTIVE FECAL SCREENINGS AT THE VETERINARY COLLEGE OF ALFORT

WHICH OF YOUR NON-HUMAN PRIMATE SPECIES WOULD YOU LIKE TO INCLUDE IN THE PROSPECTIVE STUDY?

□ *Eulemur cinereiceps*<br>□ *Eulemur (macaco) flavifrons*<br>□ *Eulemur coronatus*<br>□ *Eulemur mongoz*<br>□ *Eulemur rubriventer*<br>□ *Eulemur fulvus*<br>□ *Eulemus macaco*<br>□ *Lemur catta*<br>□ *Hapalemur alaotrensis*<br>□ *Prolemur simus*<br>□ *Propithecus coronatus*<br>□ *Varecia variegata subcinta*<br>□ *Varecia variegata*<br>□ *Galago senegalensis*	□ *Tarsius spp.*<br>□ *Lagothrix spp.*<br>□ *Saimiri sciureus*<br>□ *Saimiri boliviensis boliviensis*<br>□ *Saimiri boliviensis peruviensis*<br>□ *Saguinus bicolor*<br>□ *Saguinus midas*<br>□ *Callimico goeldii*<br>□ *Saguinus imperator*<br>□ *Saguinus oedipus*<br>□ *Leontopithecus chrysomelas*<br>□ *Leontopithecus rosalia*<br>□ *Callithrix jacchus*<br>□ *Callithrix geoffroyi*<br>□ *Cebuella pygmaea*<br>□ *Mico argentatus*<br>□ *Ateles hybridus*<br>□ *Ateles paniscus*<br>□ *Ateles fusciceps rufiventris*<br>□ *Pithecia pithecia*<br>□ *Chiropotes satanas*<br>□ *Sapajus xanthosternos*<br>□ *Cebus apella*<br>□ *Cebus capucinus*<br>□ *Alouatta spp.*	□ *Chlorocebus spp.*<br>□ *Cercopithecus roloway*<br>□ *Cercopithecus lhoesti*<br>□ *Cercopithecus hamlyni*<br>□ *Cercopithecus neglectus*<br>□ *Cercopithecus diana*<br>□ *Cercopithecus nictitans*<br>□ *Allenopithecus nigriviridis*<br>□ *Macaca tonkeana*<br>□ *Macaca Silenus*<br>□ *Macaca nemestrina*<br>□ *Macaca sylvanus*<br>□ *Papio hamadryas*<br>□ *Papio papio*<br>□ *Papio anubis*<br>□ *Theropithecus gelada*<br>□ *Lophocebus aterrimus*<br>□ *Cercocebus*	□ *Symphalangus syndactylus*<br>□ *Hylobates pileatus*<br>□ *Nomascus leucogenys*<br>□ *Nomascus siki*<br>□ *Nomascus gabriellae*<br>□ *Hylobates lar*<br>□ *Hylobates muelleri*<br>□ *Gorilla gorilla*<br>□ *Gorilla beringei*<br>□ *Pongo pygmaeus*<br>□ *Pongo abelli*<br>□ *Pan troglodytes*<br>□ *Pan paniscus*<br>□ Other

		torquatus<br>□ *Mandrillus sphinx*<br>□ *Colobus spp.*<br>□ *Procolobus spp.*<br>□ *Piliocolobus spp.*	

HOW MANY SAMPLES ARE YOU WILLING TO SEND (ideally one three-day pooled sample/species):

AT WHAT TIME OF THE YEAR COULD YOU SEND THE SAMPLES:
