# Supplementary material for: Gastrointestinal parasites in non-human primates in zoological institutions in France
Source: Parasite. 2022 Sep 20;29:43. doi: 10.1051/parasite/2022040 (PMC9487514; doi:10.1051/parasite/2022040)
Supplement: Supplementary data II: — List of non-human primate species represented in the study. [file parasite-29-43-s2.pdf]

Supplementary data II

List of non-human primate species represented in the study

Suborder	Family	Genera	Species
**Strepsirrhini**	Cheirogaleidae	*Microcebus*	*Murinus*
	Indridae	*Propithecus*	*Coronatus*
	Galagidae	*Galago*	*Senegalensis*
	Lemuridae	*Eulemur*	*Albifrons*
			Cinereiceps
			Coronatus
			flavifrons
			macaco
			mongoz
			rubriventer
		Lemur	*catta*
		Hapalemur	*alaotrensis*
		Prolemur	*simus*
		Varecia	*rubra*
			variegata
**Haplorhini – Platyrrhini**	Aotidae	*Aotus*	*griseimembra*
	Atelidae	*Alouatta*	*caraya*
		Ateles	*chamek*
			fusciceps
			geoffroyi
			hybridus
	Cebidae	*Saimiri*	*scireus*
			boliviensis
		Saguinus	*bicolor*
			imperator
			midas
			mystax
			oedipus
		Sapajus	*apella*
			xanthosternos
	Callithricidae	*Callimico*	*goeldii*
		Callithrix	*jacchus*
			geoffroyi
		Cebuella	*pygmaea*
		Leonthopithecus	*chrysomelas*
			rosalia
		Mico	*argentinus*
	Pitheciidae	*Pithecia*	*pithecia*
		Plecturocebus	*cupreus*
**Haplorhini – Catarrhini**	Cercopithecidae	*Allochrocebus*	*lhoesti*
		Cercocebus	*atys*
		Cercopithecus	*diana*
			hamlyni
			neglectus
			roloway
		Colobus	*guereza*
		Lophocebus	*aterrimus*

		Macaca	*fascicularis*
			nemestrina
			nigra
			mulatta
			silenus
			sylvanus
			tonkeana
		Mandrillus	*sphinx*
		Papio	*anubis*
			hamadryas
			papio
		Trachypithecus	*francoisi*
			gelada
	Hylobatidae	*Hylobates*	*Lar*
		Nomascus	*gabriellae*
			leucogenys
			siki
		Symphalangus	*syndactylus*
	Pongidae	*Pongo*	*pygmaeus*
	Hominidae	*Gorilla*	*gorilla*
		Pan	*troglotydes*
